# Supplementary material for: End Sequence Analysis Toolkit (ESAT) expands the extractable information from single-cell RNA-seq data
Source: Genome Res. 2016 Oct;26(10):1397–410. doi: 10.1101/gr.207902.116 (PMC5052061; doi:10.1101/gr.207902.116)
Supplement: Supplemental Material [file supp_gr.207902.116_Supp_Materials.tar.gz › ESAT-esat_v0.1/doc/html/class_jama_1_1_matrix-members.html]

ESAT: Member List


ESAT


- Main Page
- Related Pages
- Packages
- Classes
- Files

- Class List
- Class Index
- Class Hierarchy
- Class Members

All Classes Namespaces Files Functions Variables Enumerator Pages

- Jama
- Matrix

Jama.Matrix Member List

This is the complete list of members for Jama.Matrix, including all inherited members.

|  |  |  |
| --- | --- | --- |
| arrayLeftDivide(Matrix B) | Jama.Matrix |  |
| arrayLeftDivideEquals(Matrix B) | Jama.Matrix |  |
| arrayRightDivide(Matrix B) | Jama.Matrix |  |
| arrayRightDivideEquals(Matrix B) | Jama.Matrix |  |
| arrayTimes(Matrix B) | Jama.Matrix |  |
| arrayTimesEquals(Matrix B) | Jama.Matrix |  |
| chol() | Jama.Matrix |  |
| clone() | Jama.Matrix |  |
| cond() | Jama.Matrix |  |
| constructWithCopy(double[][] A) | Jama.Matrix | static |
| copy() | Jama.Matrix |  |
| det() | Jama.Matrix |  |
| eig() | Jama.Matrix |  |
| get(int i, int j) | Jama.Matrix |  |
| getArray() | Jama.Matrix |  |
| getArrayCopy() | Jama.Matrix |  |
| getColumn(int j) | Jama.Matrix |  |
| getColumnDimension() | Jama.Matrix |  |
| getColumnPackedCopy() | Jama.Matrix |  |
| getMatrix(int i0, int i1, int j0, int j1) | Jama.Matrix |  |
| getMatrix(int[] r, int[] c) | Jama.Matrix |  |
| getMatrix(int i0, int i1, int[] c) | Jama.Matrix |  |
| getMatrix(int[] r, int j0, int j1) | Jama.Matrix |  |
| getNumColumns() | Jama.Matrix |  |
| getNumRows() | Jama.Matrix |  |
| getRow(int i) | Jama.Matrix |  |
| getRowDimension() | Jama.Matrix |  |
| getRowPackedCopy() | Jama.Matrix |  |
| identity(int m, int n) | Jama.Matrix | static |
| inverse() | Jama.Matrix |  |
| isDiagonal() | Jama.Matrix |  |
| isSymmetric() | Jama.Matrix |  |
| lu() | Jama.Matrix |  |
| Matrix(int m, int n) | Jama.Matrix |  |
| Matrix(int m, int n, double s) | Jama.Matrix |  |
| Matrix(double[][] A) | Jama.Matrix |  |
| Matrix(double[][] A, int m, int n) | Jama.Matrix |  |
| Matrix(double vals[], int m) | Jama.Matrix |  |
| minus(Matrix B) | Jama.Matrix |  |
| minusEquals(Matrix B) | Jama.Matrix |  |
| norm1() | Jama.Matrix |  |
| norm2() | Jama.Matrix |  |
| normF() | Jama.Matrix |  |
| normInf() | Jama.Matrix |  |
| plus(Matrix B) | Jama.Matrix |  |
| plusEquals(Matrix B) | Jama.Matrix |  |
| print(int w, int d) | Jama.Matrix |  |
| print(PrintWriter output, int w, int d) | Jama.Matrix |  |
| print(NumberFormat format, int width) | Jama.Matrix |  |
| print(PrintWriter output, NumberFormat format, int width) | Jama.Matrix |  |
| qr() | Jama.Matrix |  |
| quantileNormalizeColumns() | Jama.Matrix |  |
| random(int m, int n) | Jama.Matrix | static |
| rank() | Jama.Matrix |  |
| read(BufferedReader input) | Jama.Matrix | static |
| set(int i, int j, double s) | Jama.Matrix |  |
| setColumn(int column, double[] vals) | Jama.Matrix |  |
| setMatrix(int i0, int i1, int j0, int j1, Matrix X) | Jama.Matrix |  |
| setMatrix(int[] r, int[] c, Matrix X) | Jama.Matrix |  |
| setMatrix(int[] r, int j0, int j1, Matrix X) | Jama.Matrix |  |
| setMatrix(int i0, int i1, int[] c, Matrix X) | Jama.Matrix |  |
| setRow(int row, double[] vals) | Jama.Matrix |  |
| setRow(int row, int[] vals) | Jama.Matrix |  |
| solve(Matrix B) | Jama.Matrix |  |
| solveTranspose(Matrix B) | Jama.Matrix |  |
| sortColumns() | Jama.Matrix |  |
| sortColumnsKeepingIndex(Matrix index) | Jama.Matrix |  |
| svd() | Jama.Matrix |  |
| times(double s) | Jama.Matrix |  |
| times(Matrix B) | Jama.Matrix |  |
| timesEquals(double s) | Jama.Matrix |  |
| toString() | Jama.Matrix |  |
| trace() | Jama.Matrix |  |
| transpose() | Jama.Matrix |  |
| uminus() | Jama.Matrix |  |
| write(String save, double[] observed) | Jama.Matrix |  |


---

Generated on Fri Aug 15 2014 15:11:06 for ESAT by  

 1.8.7
